# Current Advancement and Patient Outcomes in Reperfusion Brain Injuries After Stroke: A Comparative Analysis of Thrombolysis and Thrombectomy

**DOI:** 10.1002/brb3.70705

**Published:** 2025-08-04

**Authors:** Olobatoke Tunde Ayomide, Vishal Chavda, Bipin Chaurasia, Esther Bassey, Kanishk Dang, Henry Demian Oyoyo, Jackson T. S. Cheung, Aruni Velalakan, Odemona Glory Toluwanibukun, Nazmin Ahmed

**Affiliations:** ^1^ College of Medicine University of Lagos Lagos Nigeria; ^2^ Department of Medicine, Multispeciality, Trauma and ICCU Center Sardar Hospital Ahmedabad India; ^3^ Department of Neurosurgery College of Medical Sciences Bharatpur Nepal; ^4^ University of Uyo Uyo Nigeria; ^5^ Moldova State Univeristy Chișinău Moldova; ^6^ College of Medicine, University of Ibadan Ibadan Nigeria; ^7^ UCL Faculty of Medical Sciences London UK; ^8^ Consultant Neurosurgeon and Chairman of VNeuro Clinic Sigmaringen Germany; ^9^ Department of Paediatrics Lagos University Teaching Hospital Lagos Nigeria; ^10^ Department of Neurosurgery Ibrahim Cardiac Hospital Dhaka Bangladesh

**Keywords:** reperfusion brain injury (RBI), stroke, thrombectomy, thrombolysis

## Abstract

**Background:**

Stroke remains a leading cause of death and disability worldwide, with ischemic stroke accounting for the majority of cases. Advances in reperfusion therapies, including intravenous thrombolysis (IVT) and mechanical thrombectomy (MT), have significantly improved outcomes for acute ischemic stroke patients. However, reperfusion brain injury (RBI), a paradoxical consequence of recanalization, poses a major challenge, driven by oxidative stress, inflammation, and blood‐brain barrier disruption. This review critically examines emerging therapeutic strategies to mitigate RBI, focusing on pharmacological agents such as edaravone, NXY‐059, and tenecteplase, as well as procedural innovations in thrombectomy.

**Methods:**

This review employed a systematic search of databases such as PubMed, Cochrane Library, Embase, and Scopus using certain keywords. A comparative analysis of thrombolysis and thrombectomy was done, and emerging techniques and drugs mitigating reperfusion brain injury (RBI) were discussed.

**Results:**

Thrombolysis and thrombectomy highlight key differences in efficacy, safety, and patient selection criteria. While thrombectomy demonstrates superior outcomes in large vessel occlusions (LVOs), thrombolysis remains a cornerstone for early intervention where thrombectomy is inaccessible. Essential drugs like NXY‐059, edaravone, uric acid, *N*‐acetylcysteine, and others are changing the care of RBI after stroke. Newer thrombectomy techniques and technologies are also promising. However, the evident efficacy of these methods is still inconsistent in various patients. While thrombectomy and thrombolysis have the potential to cause post‐stroke cognitive decline, thrombectomy leads to better outcomes, but patient‐specific factors such as age, previous medical history, infarct volume, and others must be considered. Neurorehabilitation is essential for patient recovery from post‐stroke cognitive decline. Strategies such as the use of gas‐mediated therapies, pharmacological agents, stem cell therapies, antioxidant nanomedicines, and modulation of specific proteins like sirtuins are emerging treatment techniques and are promising to change the narration of RBI management and impact patient outcomes.

**Conclusion:**

The review underscores the need for precision medicine approaches, improved imaging for patient selection, and comprehensive longitudinal studies to optimize reperfusion strategies. Targeted interventions addressing oxidative damage and inflammation hold promise for reducing RBI and improving long‐term patient outcomes.

## Graphical abstract

The graphical abstract illustrates how stroke‐induced reperfusion mitigates the brain injury and induces progressive brain damage. Early therapeutic or surgical intervention can be very beneficial to reduce stroke‐reperfusion‐induced brain damage.

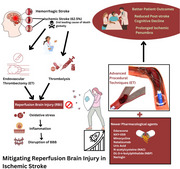

## 1 Introduction

Stroke is the second leading cause of death worldwide, responsible for 11.6% of all fatalities in 2019. Of the two primary subtypes, ischemic and hemorrhagic, ischemic stroke is the most prevalent, representing 62.4% of cases and posing significant risks of long‐term disability and mortality (GBD 2019 Stroke Collaborators 2021). This type of stroke can lead to neurological death and long‐term disabilities in adults, placing a significant burden on both healthcare systems and the economy (GBD 2019 Viewpoint Collaborators 2020; GBD 2019 Risk Factors Collaborators 2020). Key risk factors include advanced age, male sex, and comorbidities such as hypertension, diabetes, and atrial fibrillation (Palmer 2023).

Reperfusion, defined as the act of restoring blood supply, is crucial in acute stroke management. Approximately 50%–70% of ischemic stroke patients experience spontaneous reperfusion (Baird et al. 1994; Chavda, Chaurasia, Fiorindi et al. 2022). Reperfusion can also be achieved via intravenous thrombolysis (IVT) and mechanical thrombectomy (MT) as the primary treatment for acute stroke. IVT and MT have revolutionized acute ischemic stroke treatment, improving patient outcomes when administered within a specific time. IVT with alteplase (a recombinant tissue plasminogen activator [tPA]) improves outcomes in patients when administered within 4.5 h after onset, while MT of occluded large intracranial arteries improves outcomes in patients with acute ischemic stroke when performed up to 24 h after onset (Jauch et al. 2013; Turc et al. 2019; Boulanger et al. 2018).

However, reperfusion brain injury (RBI) remains a critical challenge, emerging as a paradoxical consequence of restoring blood flow. RBI develops through complex pathophysiological mechanisms. The generation of reactive oxygen species (ROS) triggers oxidative stress, initiating a cascade of cellular damage, causing direct cellular injury, leukocyte and platelet recruitment and activation, and ultimately resulting in mitochondrial dysfunction and damage to the blood‐brain barrier (BBB) (L. Lin et al. 2016; Chavda, Chaurasia, Garg et al. 2022). A newer concept in RBI is ferroptosis, a disturbance in iron metabolism leading to iron accumulation. This has the potential to generate hydroxyl species from hydrogen peroxide via a process called the Fenton reaction (Liu et al. 2024). These processes can ultimately result in brain edema, hemorrhagic transformation, substantial neuronal death, and neurological impairments (Sumii and Lo 2002). Also, zinc‐dependent metalloproteinases have been implicated in causing direct damage to the BBB (Abdul‐Muneer et al. 2016).

This review explores advancements in managing RBI, including pharmacological and procedural innovations, while comparing the efficacy and safety of thrombolysis and thrombectomy. It also discusses patient outcomes, risk factors, and future research priorities, highlighting the need for personalized strategies to optimize stroke care.

## 2 Methods

We conducted a comprehensive literature search across PubMed, Scopus, Cochrane Library, and Web of Science for peer‐reviewed articles published between 1990 and 2024. Search terms included “reperfusion injury,” “ischemic stroke,” “thrombolysis,” “thrombectomy,” “mechanical thrombectomy,” and “patient outcomes.” We included English‐language studies focusing on clinical outcomes of thrombolysis (intravenous tissue plasminogen activator [IV tPA]) and thrombectomy, with specific emphasis on stroke reperfusion therapies.

Data extraction focused on critical variables, including clinical outcomes, safety profiles, and treatment efficacy. Key metrics comprised mortality rates, functional independence, hemorrhagic transformation rates, and time to reperfusion. Studies were systematically reviewed and analyzed to provide a comprehensive assessment of current reperfusion strategies in ischemic stroke management.

### 2.1 Comparative Analysis

We conducted a qualitative synthesis comparing thrombolysis and thrombectomy, categorizing studies by treatment modality and patient outcomes. The analysis focused on differences in efficacy, safety, and long‐term recovery. Key comparative metrics included successful reperfusion rates (Thrombolysis in Cerebral Infarction [TICI] scores), symptomatic intracerebral hemorrhage (sICH) rates, and time‐to‐treatment.

The review prioritized studies with direct comparative data, systematically identifying patterns and trends across treatment modalities. Results were synthesized narratively to facilitate comprehensive analysis and discuss potential clinical implications. This approach enabled a nuanced examination of contemporary stroke reperfusion strategies, highlighting the strengths and limitations of current therapeutic interventions.

## 3 Results

### 3.1 Understanding the Mechanism and Stages of Reperfusion Brain Injuries in Different Stroke Types

RBI is an important concept in stroke care. To understand the target of the different available therapeutic drugs and procedures, it is essential to understand the mechanism and the stages of reperfusion injuries following stroke. This section will describe the mechanism of RBI through its stages, as well as patient recovery. Summarily, RBI has early, intermediate, and late stages (Algattas and Huang 2013). The early phase involves oxidative stress. Following reperfusion, there is an outburst of ROS (Yu et al. 2017). This results in oxidative stress and cellular damage. Following cellular damage, the inflammatory cascade sets in. Leukocytes, particularly, are released to the site of injury (L. Lin et al. 2016). This contributes even more to brain injury, and there is the release of inflammatory cytokines, further driving the inflammation and causing even more injury.

The intermediate phase involves platelet activation and aggregation. The reveal of the subendothelial area following endothelial injury serves as a nidus for platelet adhesions, activation, and subsequently aggregation (L. Lin et al. 2016). This mechanism can further narrow the blood vessel, contributing to the ischemia and causing further brain injury (L. Lin et al. 2016). Also, complement is activated at this phase, further contributing to inflammation and brain injury.

Finally, the late phase involves BBB disruption. Due to the chronic inflammation, the integrity of the BBB is destroyed (L. Lin et al. 2016). This can result in brain edema and hemorrhagic transformation, raising the intracranial pressure, compromising blood flow, and ultimately causing further neurological complications (L. Lin et al. 2016). Following these, there are other chronic pathophysiological mechanisms that can set in, leading to neurodegenerative diseases and possible cognitive decline. Therefore, it is essential to understand the stages of RBI and to know the stages of patient recovery and be able to administer the effective therapeutic agent. Table 1 below further summarizes and highlights the different stages and the therapeutic targets.

**TABLE 1:** Phases and reperfusion brain injury, key pathophysiological mechanisms and clinical implications.

Phase	Approximate timing	Key pathophysiological features	Clinical implications
Early phase	0–24 h post‐reperfusion	Sudden return of blood flow leads to oxidative stress (ROS burst) calcium overload mitochondrial dysfunction excitotoxicity inflammation is initiated	Target for neuroprotection, antioxidants, and free radical scavengers—time window for tPA and thrombectomy
Intermediate phase	24–72 h (3 days)	Inflammatory response intensifies: microglial activation leukocyte infiltration cytokine and chemokine release apoptosis continued BBB disruption	Target for anti‐inflammatory agents (e.g., minocycline, natalizumab)—monitor for neurological deterioration
Late phase	> 72 h to weeks/months	Transition to repair and remodeling angiogenesis neurogenesis glial scarring persistent low‐grade inflammation neurodegeneration progressive cognitive decline	Target for neurorestorative therapies, rehabilitation, and possibly immune modulation

### 3.2 New Therapies and Procedures for Reperfusion Brain Injuries

#### 3.2.1 Pharmacological Agents Targeting Reperfusion Brain Injuries

Recent pharmacological interventions have focused on mitigating reperfusion brain injuries through neuroprotective, anti‐inflammatory, and antioxidant strategies. Neuroprotective agents NXY‐059 and edaravone have been investigated for their potential to counteract oxidative stress. While the Stroke‐Acute Ischemic NXY Treatment (SAINT) trials showed inconsistent results, edaravone has effectively reduced lipid peroxidation, particularly when administered early post‐reperfusion (Shuaib et al. 2007). Edaravone has been approved in Japan and has demonstrated efficacy in reducing lipid peroxidation and improving outcomes in stroke patients, particularly when administered shortly after reperfusion (Enomoto et al. 2019).

Anti‐inflammatory agents, such as minocycline and natalizumab, have shown promise in reducing inflammation‐mediated secondary injury. Minocycline has been found to inhibit microglial activation and reduce infarct size, with some studies suggesting its efficacy when combined with thrombolysis (Myers et al. 2023; Lampl et al. 2007). Natalizumab, an antibody against α4‐integrin, has demonstrated neuroprotective effects by reducing leukocyte infiltration into the brain parenchyma, as evidenced in the ACTION trial (Khoy et al. 2020).

#### 3.2.2 Antioxidant Use in Reperfusion Brain Injury After Stroke

Antioxidants are naturally produced in the body. However, after a stroke, ROS production markedly increases to counteract their effects (Table 2). Therefore, giving antioxidants is effective in managing RBI due to their ability to inhibit ROS production, remove ROS from circulation, and break down already produced ROS (Shirley et al. 2014). Antioxidants like uric acid and *N*‐acetylcysteine are being explored to neutralize free radicals. Uric acid, when combined with tPA, potentially improves outcomes by mitigating oxidative injury. *N*‐acetylcysteine replenishes antioxidant defenses and reduces neuronal apoptosis (Arakawa and Ito 2007; Pedre et al. 2021).

**TABLE 2:** Emerging drugs targeting reperfusion brain injuries, mechanisms, and clinical evidence.

Agents	Class	Mechanism of action	Clinical evidence	Clinical status
Edaravone	Antioxidant/neuroprotective	Scavenges free radicals; reduces lipid peroxidation, protecting neurons and the blood‐brain barrier. Blocks nuclear factor erythroid 2–related factor 2/Heme oxygenase 1 (Nrf2, HO‐1) in mice.	It has been shown to improve outcomes in stroke patients when administered early post‐reperfusion (GBD 2019 Stroke Collaborators 2021).	Approved in Japan;
NXY‐059	Neuroprotective	A neuroprotective agent that reduces oxidative stress and mitigates cellular damage.	SAINT II trials showed mixed results, with limited efficacy in improving long‐term outcomes (GBD 2019 Viewpoint Collaborators 2020).	Failed in SAINT II Trial
Minocycline	Anti‐inflammatory	Inhibits microglial activation; reduces inflammation and infarct size.	Found to decrease infarct size in combination with thrombolysis in preclinical and small clinical trials (GBD 2019 Risk Factors Collaborators 2020; Palmer 2023).	Early clinical investigation
Natalizumab	Anti‐inflammatory	Blocks leukocyte infiltration by targeting α4‐integrin, reducing inflammation.	Neuroprotective effects were demonstrated in the ACTION trial, showing reduced secondary injury (Baird et al. 1994).	ACTION Trial showed limited results
Uric Acid	Antioxidant	Neutralizes free radicals; synergizes with tPA to mitigate oxidative injury.	Early studies suggest improved outcomes when combined with thrombolysis; more research is needed (Jauch et al. 2013).	Early clinical investigation
*N*‐acetylcysteine (NAC)	Antioxidant	Replenishes glutathione stores; reduces neuronal apoptosis and oxidative stress.	Emerging evidence supports its role in reducing neuronal damage in ischemia‐reperfusion injury (Turc et al. 2019).	Preclinical/Early clinical investigation
DL‐3‐*n*‐butylphthalide (NBP)	Neuroprotective	Reduces mitochondrial apoptosis and oxidative stress; improves cerebral microcirculation.	Shown to reduce cerebral infarction size and improve neurological outcomes in preclinical studies (Boulanger et al. 2018).	Approved in Japan for clinical use.
Naringin	Antioxidant/anti‐inflammatory	A flavonoid that alleviates inflammation and oxidative stress during reperfusion injury.	Preclinical studies indicate protective effects against ischemia‐reperfusion injury in animal models (L. Lin et al. 2016).	Preclinical
Ferroptosis Inhibitors	Antioxidants/anti‐inflammatory	It inhibits the iron‐dependent lipid peroxidation pathway	Ferrostatin‐1 has been identified as a potential agent (Liu et al. 2024).	Preclinical

Recent studies have introduced new neuroprotective agents such as dl‐3‐*n*‐butylphthalide (NBP), which significantly reduces cerebral infarction size and improves neurological outcomes by inhibiting mitochondrial apoptosis pathways. Naringin, a flavonoid compound, has also shown protective effects against ischemia‐reperfusion injury by alleviating inflammation and oxidative stress (Wang et al. 2021). Additionally, emerging research on ferroptosis inhibitors indicates a novel approach to mitigating reperfusion injury by targeting oxidative damage pathways (T. L. Zhang et al. 2023; L. Zhang, Bai, et al. 2024). Innate mechanisms, including genes and chemical substances targeting this pathway of stroke pathology, have been discussed (Tian et al. 2024). Recent studies have identified ferrostatin‐1 as a potential agent that can block this pathway and improve patient care (L. Zhang, Luo, et al. 2024; Miotto et al. 2020). But its mechanism of action is still currently being explored, and there is no clinical evidence so far.

#### 3.2.3 Procedural Innovations and Adjunctive Therapies

Advancements in imaging and procedural techniques have significantly improved the management of reperfusion brain injuries. Advanced imaging modalities, such as perfusion‐weighted MRI and CT angiography, allow for the accurate delineation of the ischemic penumbra and infarct core, guiding therapeutic decisions and improving patient selection for reperfusion therapies (Baird et al. 1994; Albers et al. 2018).

The use of retriever stents has also proven to be faster at ensuring endovascular recanalization and mitigating side effects such as hemorrhage and stenosis, which are usually seen in temporary and permanent stents (Brekenfeld et al. 2011). Neurovascular stents and aspiration devices have revolutionized MT. Devices like Solitaire and Trevo have demonstrated high rates of successful recanalization and improved functional outcomes compared to older technologies (Goyal et al. 2016). Innovations such as balloon‐guided catheters and distal access catheters have further reduced the risks of distal embolization and improved procedural safety (Berkhemer et al. 2015).

**FIGURE 1:**
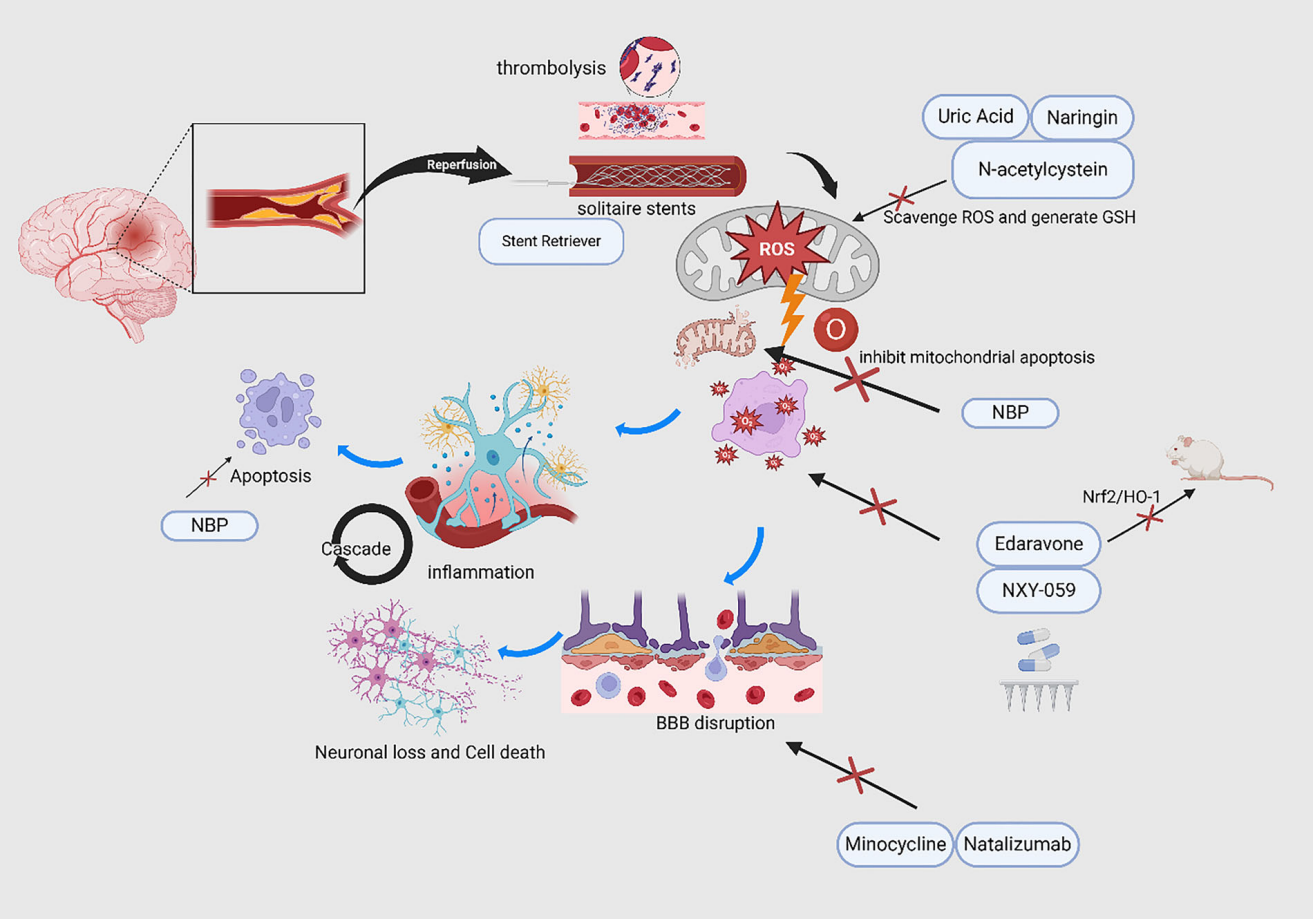
A Schematic Illustration of RBI After Stroke and Important Drugs and Their Targets. Reperfusion can be achieved via thrombolysis/thrombectomy. The generation of ROS remains the hallmark of RBI. Inflammatory cascade, mitochondrial apoptosis, BBB disruption, neuronal loss, and cell death are all important sequelae, among others. BBB, blood‐brain barrier; GSH, glutathione; HO‐1, heme oxygenase 1. NBP, DL‐3‐*n*‐butylphthalide; Nrf2, nuclear factor erythroid 2‐related factor 2; RBI, reperfusion brain injury; ROS, reactive oxygen species. Created in https://BioRender.com.

The DEFUSE 3 and DAWN trials have underscored the utility of these imaging and stenting techniques in extending the therapeutic window for thrombectomy up to 24 h post‐stroke onset (Albers et al. 2018; Nogueira et al. 2018). Adjunctive therapies, including therapeutic hypothermia and ischemic post‐conditioning, are being investigated to attenuate reperfusion injuries. Therapeutic hypothermia, by reducing cerebral metabolism and stabilizing the BBB, has shown neuroprotective effects in preclinical studies; however, clinical trials have yielded mixed results regarding its efficacy (Collins and Samworth 2008). Ischemic post‐conditioning has demonstrated promise in animal models by reducing infarct size through intermittent blood flow interruptions during reperfusion (Kleinbongard et al. 2016). Figure 1 summarizes these drugs, techniques, and their targets.

### 3.3 Impact of Thrombolysis and Thrombectomy on Reperfusion Brain Injuries

#### 3.3.1 Mechanisms of Thrombolysis and Thrombectomy

Thrombolysis, typically with tPA, works by activating plasminogen to plasmin, which subsequently breaks down fibrin within the thrombus, restoring cerebral blood flow. However, its utility is limited by a narrow therapeutic window of 4.5 h and an increased risk of hemorrhagic transformation (Hacke et al. 1999; Emberson et al. 2014). Alternatives like tenecteplase, a genetically modified variant of tPA, have shown similar efficacy with potentially lower bleeding risks (Emberson et al. 2014; Liang et al. 2023). MT, involving devices such as stent retrievers or aspiration catheters, physically extracts the clot, offering a higher rate of complete recanalization, particularly in patients with large vessel occlusions (LVOs) (Goyal et al. 2016). The extended window for thrombectomy, as evidenced in the DAWN and DEFUSE 3 trials, has allowed for treatment up to 24 h after symptom onset, significantly broadening the eligible patient population (Albers et al. 2018; Nogueira et al. 2018).

#### 3.3.2 Effects on Reperfusion Injury

Reperfusion injury is characterized by oxidative stress, inflammation, and disruption of the BBB, leading to exacerbated neuronal damage. Thrombolysis can increase the risk of hemorrhagic transformation, particularly in patients with pre‐existing cerebrovascular abnormalities or those presenting with hyperglycemia (Jauch et al. 2013; Sun et al. 2018). New thrombolytic agents, such as desmoteplase and modified tPA, are being developed to reduce these risks while maintaining fibrinolytic efficacy (Patel et al. 2014). Thrombectomy can also cause reperfusion injury, especially in cases of prolonged ischemia. Strategies to mitigate these effects include using neuroprotective agents administered intra‐arterially during the procedure and controlled reperfusion techniques (Zhou et al. 2023). The combination of thrombectomy with thrombolysis may increase the risk of hemorrhagic complications, necessitating careful patient selection and post‐procedural monitoring (Goyal et al. 2016).

#### 3.3.3 Comparative Analysis of Thrombolysis and Thrombectomy

Thrombectomy has been shown to result in superior outcomes compared to thrombolysis alone, particularly in patients with LVOs. The HERMES collaboration—a meta‐analysis of individual patient data from five randomized trials—demonstrated that thrombectomy resulted in significantly better functional outcomes and lower mortality compared to thrombolysis (Goyal et al. 2016). The advantages of thrombectomy are particularly evident in patients who present beyond the traditional thrombolysis window, as highlighted by the DAWN and DEFUSE 3 trials (Albers et al. 2018; Nogueira et al. 2018). Thrombolysis remains essential for patients presenting within 4.5 h of stroke onset and for those without access to thrombectomy‐capable centers. However, developing novel thrombolytic agents like tenecteplase and improved imaging techniques are expanding their role even for patients who may benefit from thrombectomy (Emberson et al. 2014).

#### 3.3.4 Patient Risk Factors for the Development of RBI and Outcomes

Several factors predispose patients to reperfusion brain injuries. Age is a significant risk factor; older patients are more likely to experience hemorrhagic transformation and poorer functional recovery following reperfusion therapy (Avula et al. 2024). Comorbid conditions, including hypertension, diabetes, and atrial fibrillation, increase adverse outcomes by exacerbating inflammatory and oxidative responses during reperfusion (Jauch et al. 2013; Sun et al. 2018). Male sex has also been implicated as a significant predisposing factor for RBI and poorer patient outcomes (Kasemsap et al. 2022; Luqman et al. 2023).

Large infarct volumes and cerebral microbleeds detected through susceptibility‐weighted imaging are associated with higher risks of hemorrhagic transformation following reperfusion therapy (Nagaraja et al. 2021; Chen et al. 2022). Advanced imaging techniques such as perfusion‐weighted MRI and CT perfusion are increasingly used to assess tissue viability and guide therapeutic decisions, potentially reducing risks associated with reperfusion injuries (Broocks and Meyer 2023; Copen et al. 2011).

Patient outcomes after reperfusion therapy are influenced by the risk factors previously discussed and other factors such as the speed of revascularization, the extent of initial brain injury, and complications like hemorrhagic transformation or cerebral edema. Early administration of thrombolysis has been associated with better short‐term neurological recovery; however, long‐term outcomes may be limited by risks such as hemorrhagic complications, especially in older patients or those with large infarcts (Hacke et al. 1999; Emberson et al. 2014). Thrombectomy has been associated with improved functional recovery and reduced mortality rates, particularly in patients with LVOs. The MR CLEAN and SWIFT PRIME trials demonstrated significant improvements in outcomes at 90 days post‐treatment, with many patients achieving independence in daily activities (Goyal et al. 2016; Berkhemer et al. 2015). Long‐term prognosis after thrombectomy is generally favorable; however, some patients may experience residual deficits or delayed complications such as post‐stroke seizures or cognitive impairment (Jauch et al. 2013). The clinical advantage of the use of thrombectomy following anticoagulation is also being explored in distal medium vessel occlusion (DMVO) and LVO, but the results so far are still not significant in terms of patient improvement (Salim et al. 2024).

Additionally, different measurement scores have been discovered and validated to predict patient outcomes clinically. The ASPECTS (Alberta Stroke Program Early CT Score) is a quantitative score that has been adapted to measure the degree of acute brain infarction using the early CT scan findings (Pexman et al. 2001). It is usually measured in Hounsfield units (HU). Studies have shown that an HU score ratio between 0.94 and 0.96 has the highest correlation with the final infarct volume. Although further validation is still needed (Mokin et al. 2017). This score has been shown to be particularly essential in predicting the outcomes of LVO ischemic stroke needing thrombectomy (Alexandre et al. 2024). The National Institutes of Health Stroke Scale (NIHSS) is another important scoring system that can be used for predicting stroke outcomes. Evidence has shown that a higher score is associated with poorer patient outcomes (Luqman et al. 2023).

## 4 Discussion

The comparison of thrombolysis with thrombectomy in the management of reperfusion brain damage following ischemic stroke reveals significant differences in their processes, therapeutic windows, and patient outcomes. Thrombolysis, usually conducted with tPA, is most effective within 4.5 h after the onset of a stroke (Powers et al. 2019). This technique operates by dissolving the thrombus and reinstating blood flow, although it entails considerable danger of hemorrhagic transformation, particularly in individuals with extensive infarcts, hypertension, or hyperglycemia (Khoy et al. 2020). Disrupting the BBB during reperfusion may lead to further neuronal injury via oxidative stress and inflammatory responses, hence delaying recovery (Sumii and Lo 2002; Khoy et al. 2020).

Conversely, MT, which entails the physical extraction of clots from major vascular occlusions (LVOs), provides an extended therapeutic window, lasting up to 24 h post‐stroke in meticulously chosen patients (Shuaib et al. 2007). Thrombectomy demonstrates higher recanalization rates and improved functional results relative to thrombolysis, especially in patients with LVOs (Shuaib et al. 2007; Lees et al. 2006). The findings of extensive trials, including DEFUSE 3 and DAWN, substantiate the advantages of thrombectomy in prolonging the therapy window beyond the conventional parameters of thrombolysis (Turc et al. 2019; Lees et al. 2006).

Nonetheless, both methodologies possess intrinsic dangers associated with reperfusion injury. Thrombectomy, although superior for recanalization, may nevertheless lead to reperfusion‐related problems such as oxidative damage, BBB disruption, and hemorrhagic transformation, especially in instances of protracted ischemia (Sumii and Lo 2002; Shuaib et al. 2007; Lees et al. 2006). Moreover, many studies indicate that the amalgamation of thrombolysis and thrombectomy may elevate the risk of sequelae, including hemorrhagic transformation, highlighting the necessity for meticulous patient selection and post‐procedural surveillance (Sumii and Lo 2002; Myers et al. 2023; Amaro and Chamorro 2016).

Thrombectomy generally provides superior outcomes for a wider array of patients; nonetheless, thrombolysis is essential for those who present early or lack access to thrombectomy‐capable facilities (Shuaib et al. 2007; Powers et al. 2019). The selection of these techniques should be determined by patient‐specific considerations, such as stroke severity, comorbidities, and the timing of intervention (Khoy et al. 2020; Powers et al. 2019).

### 4.1 Therapeutic Advances and Strategic Insights

Substantial progress in thrombolysis and thrombectomy has enhanced the treatment of reperfusion brain injuries. In thrombolysis, novel thrombolytic drugs like tenecteplase present a prospective alternative to alteplase, exhibiting a comparable effectiveness profile while potentially reducing the risk of hemorrhagic consequences (Amaro and Chamorro 2016). This agent's extended half‐life and ease of administration render it a preferable choice in scenarios where prompt treatment is essential (Powers et al. 2019; Amaro and Chamorro 2016). Furthermore, pharmaceutical innovations aimed at the causes of reperfusion injury have demonstrated promise in mitigating oxidative stress and inflammation. Neuroprotective drugs, including NXY‐059 and edaravone, have been studied for their potential to mitigate oxidative damage during reperfusion; however, clinical outcomes have been inconsistent (Myers et al. 2023). Edaravone has demonstrated potential in Japan, and it works by diminishing lipid peroxidation, an injury‐causing mechanism used by ROS. It also safeguards against BBB disturbance (Wang et al. 2021; T. L. Zhang et al. 2023). Whereas, NXY‐059 is inefficient in the treatment of acute ischemic stroke according to the SAINT II trial, as there is no efficacy with the use of the drug compared to placebo (Shuaib et al. 2007; Lees et al. 2006). These findings seemed to have dashed our hope of a new drug to target acute ischemic stroke. However, Savitz and Schäbitz (2008) in their publication, argued a possible neutralization effect of tPA on NXY‐059, although there is not enough evidence given the study methodology. Also, the inability of the SAINT II trial study to clearly categorize the acute ischemic patients into either white or grey matter stroke is another important reason why NXY‐059 might have proven ineffective (Savitz and Schäbitz 2008). Perhaps, future studies can attempt to better classify participants, as NXY‐059 targets free radicals; the effect of these free radicals differs in various parts of the brain (Savitz and Schäbitz 2008; Diener et al. 2008).

Additionally, natalizumab is not cost‐effective. In a double‐blinded stroke trial, infarct volume 9 h post‐stroke is not reduced (Elkins et al. 2017). Compared to a placebo, it has similar adverse effects and outcomes. Meaning that more investigation is needed to properly categorize the group of patients and the duration for which the medication is most effective (Elkins et al. 2017). On the other hand, edaravone has been shown to be cost‐effective when compared to other medications targeted at acute ischemic stroke care. In China, it was shown to be cost‐effective when compared to the NBP arm (Li et al. 2024). Although both drugs have the same cost implications, patients taking edaravone gained an additional 0.1615 quality‐adjusted life years. It was also shown to be associated with the reduction of the total cost of stroke care compared to ozragel therapy at a hospital in Japan (Enomoto et al. 2019; Shinohara et al. 2009). It was, therefore, conclusively stated to be the important drug of choice to be considered in a low socio‐economic environment where there is a need to provide effective care at a low cost.

MT has experienced significant technological advancements. Devices such as stent retrievers and aspiration catheters have transformed the treatment, yielding increased recanalization rates and improved functional recovery (Turc et al. 2019; Shuaib et al. 2007). Innovations like balloon‐guided catheters and distal access catheters have mitigated the dangers of distal embolization during thrombectomy, enhancing safety and efficacy (Shuaib et al. 2007). Moreover, sophisticated imaging techniques like perfusion‐weighted MRI and CT angiography have improved the accuracy of patient selection, facilitated superior identification of the ischemic penumbra, and refined the decision‐making process concerning candidates most likely to benefit from reperfusion therapies (Baird et al. 1994; Wang et al. 2021; Lees et al. 2006).

Adjunctive therapies have garnered interest for their capacity to alleviate reperfusion brain damage. Therapeutic hypothermia has been investigated as a method to diminish cerebral metabolism and stabilize the BBB, although clinical outcomes have been inconsistent (Arakawa and Ito 2007). Ischemic post‐conditioning, characterized by intermittent cessation of blood flow after reperfusion, has shown neuroprotective effects in animal models but necessitates additional research in human environments. These improvements highlight the significance of a patient‐specific approach to stroke management, wherein treatment is adapted according to individual risk factors, timing of presentation, and available resources (GBD 2019 Risk Factors Collaborators 2020; Arakawa and Ito 2007).

### 4.2 Clinical Implications and Patient Care

From a therapeutic perspective, the management of reperfusion brain injuries necessitates a meticulous equilibrium between the advantages of revascularization and the hazards of intensifying neuronal damage. The restricted therapeutic window and heightened risk of hemorrhagic transformation in thrombolysis, especially among older patients or those with comorbidities like diabetes and hypertension, require meticulous patient selection (Khoy et al. 2020). Clinicians must evaluate alternate medications such as tenecteplase, which may present enhanced safety profiles, and must remain attentive to monitoring patients for indications of hemorrhagic complications, especially in the hours after therapy (Amaro and Chamorro 2016).

MT, despite its extended treatment window and elevated recanalization rates, is not devoid of hazards. Reperfusion damage, characterized by oxidative stress and BBB disruption, continues to be a concern, particularly in patients with extended ischemic or substantial infarct volumes (Sumii and Lo 2002; Shuaib et al. 2007). The use of neuroprotective medications during the surgery, along with regulated reperfusion procedures, may alleviate these concerns (Myers et al. 2023; T. L. Zhang et al. 2023).

Both therapies should include improved imaging tools in clinical practice to enhance patient selection for reperfusion therapy, especially for those with salvageable brain tissue within the ischemic penumbra. Imaging can aid in predicting the risk of hemorrhagic transformation by identifying microbleeds or evaluating the degree of BBB disruption, thereby assisting physicians in determining the most suitable treatment for each patient (Enomoto et al. 2019; Wang et al. 2021).

Furthermore, the management of reperfusion brain injuries necessitates vigilant post‐procedural surveillance to detect early indicators of problems, including hemorrhagic transformation, cerebral edema, or seizures (Sumii and Lo 2002; Khoy et al. 2020; Wang et al. 2021). Particular focus must be directed towards high‐risk populations, including elderly individuals, patients with extensive infarcts, or those with comorbidities such as hypertension, diabetes, or atrial fibrillation, as these groups exhibit increased vulnerability to negative outcomes. Clinicians must examine the time and combination of treatments, as the conjunction of thrombolysis and thrombectomy may elevate the risk of hemorrhagic consequences, hence requiring personalized treatment strategies (Khoy et al. 2020; T. L. Zhang et al. 2023; Powers et al. 2019; Savaliya et al. 2024a).

A crucial element of patient care is the incorporation of novel medications that address specific mechanisms of reperfusion injury. Agents that mitigate oxidative stress, such as edaravone or NXY‐059, may be utilized with reperfusion therapy to diminish neuronal injury, especially in high‐risk patients, necessitating appropriate patient selection (Myers et al. 2023; T. L. Zhang et al. 2023). Moreover, anti‐inflammatory agents such as minocycline or natalizumab have demonstrated potential in mitigating secondary damage resulting from leukocyte infiltration and microglial activation, offering an alternative strategy for enhancing patient outcomes. These observations underscore the need for a comprehensive strategy for managing reperfusion brain injuries, wherein meticulous patient selection, prompt intervention, and use of adjuvant medications might improve clinical outcomes (Sumii and Lo 2002; Wang et al. 2021).

### 4.3 Post‐Stroke Cognitive Impairment Following Thrombectomy and Thrombolysis

#### 4.3.1 Mechanism of Cognitive Impairment Following Brain Reperfusion: Thrombectomy Versus Thrombolysis

There is several evidence describing the mechanism of cognitive decline following brain reperfusion after a stroke (Xia et al. 2024; Alawieh et al. 2020; Yang et al. 2024; B. Lin et al. 2024). Reperfusion after ischemic stroke leads to a decline in neuronal bioenergy, primarily due to mitochondrial dysfunction. The CDK9/p53/VDAC signaling pathway has been implicated as a significant factor in this process. There is a downregulation of the mitochondrial voltage‐dependent anion channels (VDACS) due to triggers coming from the p53 gene. This ultimately can lead to the depletion of adenosine triphosphate (ATP). This eventually contributes to progressive cognitive decline and possible neuropsychiatric symptoms (Xia et al. 2024).

Neuroinflammation has also been noted to play a significant role in post‐stroke cognitive decline. Even after successful reperfusion, microglial cells, via phagocytosis and using a complement‐dependent mechanism, continue to cause neuronal tissue death and loss. They cause synaptic density decline. Targeted inhibition of the complement system, such as through the use of B4Crry, has been shown to reduce microgliosis and improve cognitive outcomes (Kwon and Koh 2020).

### 4.4 Comparative Cognitive Outcomes: Thrombectomy Versus Thrombolysis

Despite the various significant pieces of evidence supporting cognitive impairment following reperfusion via thrombolysis or thrombectomy. These procedures can lead to improved cognitive outcomes (Tawil and Muir 2017). Thrombectomy, in particular, has been associated with favorable cognitive outcomes when compared to thrombolysis due to its ability to restore blood flow in LVO within the shortest time possible, thereby minimizing brain damage (Roaldsen et al. 2021; Humphrey et al. 2024). Thrombolysis, on the other hand, has shown mixed results, especially in patients with pre‐existing cognitive impairments, where outcomes tend to be less favorable (Ramnarine et al. 2023).

The ESCAPE trial demonstrated that endovascular thrombectomy (EVT) significantly improved cognitive outcomes across multiple tests, including the Montreal Cognitive Assessment (MoCA) and the Boston Naming Test, with higher odds of favorable outcomes compared to standard care (Joundi et al. 2024). A similar study, which aimed to identify biomarkers of predictive cognitive impairment post‐thrombectomy, showed the potential of targeted rehabilitation to improve cognitive outcomes post‐stroke. Patient‐specific factors such as infarct size, total infarct volume, and the degree of white matter involvement have been found to have the potential to worsen cognitive outcomes (Ospel et al. 2024). This finding supports the role of timely EVT in reducing the severity of brain damage post‐stroke and subsequently minimizing the rate of cognitive decline. Shorter reperfusion times in EVT are associated with better cognitive outcomes, as demonstrated by the inverse relationship between time to recanalization and cognitive scores in the ACE‐R assessment (Costa Novo et al. 2024). Also, factors such as prior cognitive problems such as dementia before stroke and female sex have been implicated in the severity (Ramnarine et al. 2023). Females, particularly those who are widows or with low socioeconomic status, are at risk of post‐stroke cognitive decline compared to men (Dong et al. 2020).

Thrombolysis, often combined with standard medical care, showed less pronounced cognitive benefits compared to thrombectomy. However, it remains a critical treatment option, especially when thrombectomy is not feasible. Humphrey et al. (2024) found that patients treated with endovascular clot retrieval (ECR) had better cognitive outcomes than those receiving standard medical care, which included thrombolysis, suggesting a superior benefit of thrombectomy in cognitive recovery.

Invariably, it can be concluded that while thrombectomy shows promising results in enhancing cognitive function post‐stroke, it is crucial to consider individual patient factors, such as sex, prior medical history, socioeconomic status, infarct characteristics, and reperfusion times, to optimize outcomes. Thrombolysis remains a valuable treatment, particularly when thrombectomy is not an option, but its cognitive benefits may be less pronounced.

Table 3 below summarizes the characteristic comparisons between thrombolysis and thrombectomy while critically analyzing their differences and key clinical considerations.

**TABLE 3:** Comparative Analysis of Thrombectomy Versus Thrombolysis.

Feature	Thrombolysis (tPA)	Thrombectomy	Key Considerations
Time window	Up to 4.5 h	Up to 24 h (select cases)	Thrombectomy extends the treatment window, benefiting late presenters.
Mechanism	Enzymatic clot dissolution	Physical clot retrieval via catheter	Different mechanisms influence treatment selection.
Effectiveness	Moderate in small thrombi; limited in large vessel occlusions (LVOs)	Highly effective in LVOs; direct clot removal ensures better recanalization	Thrombectomy has higher success in cases of large artery occlusion.
Reperfusion quality	Partial or incomplete in some cases	Higher rates of complete reperfusion	Incomplete reperfusion in thrombolysis may contribute to cognitive decline.
Adverse events	Higher risk of hemorrhagic transformation and excitotoxic injury	Risk of vessel perforation, embolization, or distal clot migration	Adverse events vary based on patient selection and technique.
Cognitive impact	Possible subtle long‐term executive dysfunction due to delayed ischemic injury	Potential microvascular dysfunction and oxidative stress contributing to delayed cognitive decline	Cognitive impairment can manifest differently based on intervention type.
Accessibility	Available in most stroke centers	Requires specialized stroke centers with interventional capabilities	Accessibility gaps may influence treatment decisions.
Combination therapy	Sometimes used before thrombectomy (bridging therapy)	May improve outcomes but increases hemorrhage risk	The optimal approach remains under investigation.
Future considerations	Development of safer fibrinolytics	Enhanced imaging for better patient selection	Precision medicine approaches may refine patient eligibility.

### 4.5 Neurorehabilitation Strategies for Cognitive Recovery after Reperfusion Therapy

Newer studies have highlighted the impact of neurorehabilitation in addressing cognitive decline after stroke by leveraging various innovative approaches. Recently, the effectiveness of neurorehabilitation techniques such as multidomain cognitive training and transcranial direct current stimulation (tDCS) has been documented, as well as the importance of neuroplasticity in rehabilitation strategies. These methods aim to improve cognitive functions and overall quality of life for stroke survivors.

Multidomain cognitive training is a neurorehabilitation therapy that involves activities such as object recognition, fear recognition, and navigation of various tasks to improve brain function (Mehla et al. 2023). Evidence has shown that the use of tele‐neurorehabilitation using a multidomain cognitive training approach significantly improved working memory and language abilities in stroke patients (Contrada et al. 2024). This not only impacts the patient but also has the potential to alleviate caregivers’ burdens (Contrada et al. 2024).

Also, tDCS has emerged as a promising neurorehabilitation technique that enhances neuroplasticity via neuromodulation, potentially improving cognitive functions post‐stroke. It particularly leads to the reorganization of the neurons and synapses, which are very important in the process of cognitive recovery (Sloane and Hamilton 2024). The process of neuroplasticity is essentially fundamental in stroke recovery. While these advancements in neurorehabilitation show promise, challenges remain, such as the variability in patient responses and the need for more extensive clinical trials to validate these approaches (Gunduz et al. 2023). Personalized rehabilitation strategies are showing better outcomes (Du et al. 2023).

### 4.6 Future Research Directions

Considerable progress has been made in reperfusion therapies; however, some domains remain inadequately investigated, offering prospects for future inquiry. A significant challenge in stroke care is forecasting which patients are most susceptible to reperfusion brain damage. Present risk indicators, including age, comorbidities, and infarct size, offer some direction; nevertheless, new specific biomarkers are required to enhance patient stratification. Investigating genetic and molecular markers may facilitate the identification of patients predisposed to complications such as hemorrhagic transformation or cerebral edema, enabling doctors to customize therapies more efficiently (Sumii and Lo 2002; Khoy et al. 2020; Wang et al. 2021).

Additional research is required to enhance the application of neuroprotective drugs with reperfusion treatments. Although several medications, such as edaravone, exhibit potential, the variable outcomes from clinical trials suggest that further research is necessary to ascertain the most efficacious agents, doses, and time for administration (Myers et al. 2023; Pedre et al. 2021). The advancement of novel pharmacological therapies, including ferroptosis inhibitors, signifies a promising study domain. These drugs particularly target the oxidative damage pathways associated with reperfusion injury and may provide a novel therapeutic approach for mitigating neuronal damage (Wang et al. 2021; T. L. Zhang et al. 2023).

Future investigations should focus on enhancing procedural approaches in MT. Although thrombectomy has transformed stroke management, enhancing the operation to minimize distal embolization, vascular damage, and reperfusion injury continues to be a focus. Improvements in device technology, such as optimally designed stent retrievers and aspiration catheters, may augment the safety and effectiveness of thrombectomy (Sumii and Lo 2002; Shuaib et al. 2007; Pedre et al. 2021). Furthermore, optimizing supplementary therapy, including therapeutic hypothermia or ischemic post‐conditioning, may enhance neuroprotection; nevertheless, more substantial clinical data is required to endorse their routine application (Arakawa and Ito 2007).

Ultimately, longitudinal research on patient outcomes following reperfusion treatment is crucial. Although short‐term recovery is often assessed by functional independence at 90 days, further investigation is required to comprehend the long‐term cognitive and neurological consequences of reperfusion brain injuries. Comprehending the postponed effects of reperfusion, like post‐stroke seizures, cognitive deterioration, or depression, is essential for enhancing the overall quality of life in stroke survivors (Albers et al. 2018). Furthermore, subsequent studies should investigate the integration of personalized medicine into stroke therapy. The growing utilization of modern imaging and genetic profiling is rendering the customization of reperfusion techniques to specific patient features increasingly viable. For instance, research examining the influence of pre‐existing diseases, such as diabetes or hypertension, on reperfusion injury may enhance treatment regimens. By comprehending the responses of certain patient subgroups to thrombolysis or thrombectomy, doctors can enhance outcome predictions and modify therapy accordingly. Furthermore, investigating the interplay between individual patient characteristics and novel therapeutic agents, such as neuroprotective medications or anti‐inflammatory therapy, can enhance recovery and reduce problems (Albers et al. 2018; Copen et al. 2011; Nagaraja et al. 2021; Chen et al. 2022; Broocks and Meyer 2023; Copen et al. 2011; Savaliya et al. 2024b).

### 4.7 Overview of Emerging Trends and Potential Breakthroughs in RBI Management

Emerging trends in the management of reperfusion brain injury focus on innovative neuroprotective strategies that aim to mitigate the damage caused by ischemia‐reperfusion events and prevent post‐stroke cognitive decline. Strategies such as the use of gas‐mediated therapies, pharmacological agents, stem cell therapies, antioxidant nanomedicines, and modulation of specific proteins like sirtuins are gaining attraction and are currently being explored using animal models. Each of these approaches targets a particular aspect of the mechanism of reperfusion brain injury following a thrombectomy or thrombolysis. The next few paragraphs briefly highlight some of these emerging trends to show what the future of reperfusion injury management looks like.

Gas‐mediated and pharmacological therapies are newer techniques currently being explored. Preclinical studies have attempted the use of gases such as nitric oxide, hydrogen, and xenon to combat RBI. These gases have shown promise in reducing neuronal cell loss in animal models (Marasini and Jia 2024). Marasini et al. in their study, equally described the role of stem cell therapies in combating RBI. They highlighted how stem cell‐based approaches are being explored for their potential to promote neurological recovery post‐cardiac arrest. However, these therapies are still in the preclinical stage, with limited studies showing subtle benefits (Marasini and Jia 2024).

The use of antioxidant nanomedicines is also currently being explored, and it is a novel approach to delivering antioxidants to the ischemic penumbra. They have the potential to overcome the traditional antioxidants, which have low bioavailability and numerous side effects (Hou and Brenner 2024).

The Sirtuin protein family has been identified to have the potential to target the inflammatory mechanism and oxidative stress that usually follow reperfusion. Their mechanism is currently being explored and not well understood, but they have the potential to be used as a therapeutic intervention for RBI (Ye et al. 2024). *N*, *N*‐dimethyltryptamine (DMT) is also a newer drug currently being explored. Its underlying mechanism is not very clear for now, but it is an endogenous agonist of the Sigma‐1 receptor with the potential to improve mitochondrial function and reduce cellular stress, suggesting its possible use in RBI management (Kovacs et al. 2024).

## 5 Conclusion

The comparative examination of thrombolysis and thrombectomy reveals substantial progress in the treatment of reperfusion brain damage following a stroke. Thrombectomy, characterized by its prolonged therapeutic window and elevated recanalization rates, has demonstrated notable efficacy in addressing major vascular occlusions, but thrombolysis continues to be a crucial method for prompt intervention. Both treatments, however, entail hazards of reperfusion injury, including oxidative stress, hemorrhagic transformation, and BBB disruption, highlighting the necessity for individualized treatment techniques.

Therapeutic advancements, encompassing innovative thrombolytic agents, neuroprotective medications, and procedural innovations, possess the capacity to enhance results and reduce reperfusion damage. Clinical considerations must account for individual risk factors, including age, comorbidities, and the degree of brain damage, to ensure that the advantages of reperfusion surpass the associated dangers.

Future research ought to concentrate on the development of biomarkers for enhanced patient stratification, the optimization of neuroprotective medications, and the refinement of procedural procedures in thrombectomy. Longitudinal studies on patient outcomes and the socioeconomic implications of stroke therapies are essential for informing future guidelines and guaranteeing that the most efficacious treatments are available to all patients.

## Author Contributions

**Olobatoke Tunde Ayomide**: conceptualization, investigation, writing – original draft. **Vishal Chavda**: methodology, supervision. **Bipin Chaurasia**: validation, visualization, writing – review and editing. **Esther Bassey**: validation. **Kanishk Dang**: formal analysis. **Henry Demian Oyoyo**: data curation. **Jackson T. S. Cheung**: Supervision. **Aruni Velalakan**: formal analysis. **Odemona Glory Toluwanibukun**: methodology. **Nazmin Ahmed**: visualization.

## Ethics Statement

The authors have nothing to report.

## Conflicts of Interest

The authors declare no conflicts of interest.

## Peer Review

The peer review history for this article is available at https://publons.com/publon/10.1002/brb3.70705.

## Data Availability

Data availability is not applicable to this article, as no new data were created or analyzed in this study.

## References

[brb370705-bib-0001] Abdul‐Muneer, P. M. , B. J. Pfister , J. Haorah , and N. Chandra . 2016. “Role of Matrix Metalloproteinases in the Pathogenesis of Traumatic Brain Injury.” Molecular Neurobiology 53, no. 9: 6106–6123. 26541883 10.1007/s12035-015-9520-8 PMC9470225

[brb370705-bib-0002] Alawieh, A. M. , E. F. Langley , W. Feng , A. M. Spiotta , and S. Tomlinson . 2020. “Complement‐Dependent Synaptic Uptake and Cognitive Decline After Stroke and Reperfusion Therapy.” Journal of Neuroscience 40, no. 20: 4042–4058. 32291326 10.1523/JNEUROSCI.2462-19.2020 PMC7219298

[brb370705-bib-0003] Albers, G. W. , M. P. Marks , S. Kemp , et al. 2018. “Thrombectomy for Stroke at 6 to 16 H With Selection by Perfusion Imaging.” New England Journal of Medicine 378, no. 8: 708–718. 29364767 10.1056/NEJMoa1713973 PMC6590673

[brb370705-bib-0004] Alexandre, A. M. , M. Monforte , V. Brunetti , et al. 2024. “Baseline Clinical and Neuroradiological Predictors of Outcome in Patients With Large Ischemic Core Undergoing Mechanical Thrombectomy: A Retrospective Multicenter Study.” International Journal of Stroke 19, no. 7: 779–788. 38546177 10.1177/17474930241245828 PMC11298113

[brb370705-bib-0005] Algattas, H. , and J. H. Huang . 2013. “Traumatic Brain Injury Pathophysiology and Treatments: Early, Intermediate, and Late Phases Post‐Injury.” International Journal of Molecular Sciences 15, no. 1: 309–341. 24381049 10.3390/ijms15010309 PMC3907812

[brb370705-bib-0006] Amaro, S. , and Á. Chamorro . 2016. “Should Uric Acid be Administered Alongside Thrombolysis for Stroke Patients?.” Expert Review of Cardiovascular Therapy 14, no. 4: 407–409. 26788663 10.1586/14779072.2016.1144470

[brb370705-bib-0007] Arakawa, M. , and Y Ito . 2007. “ *N*‐Acetylcysteine and Neurodegenerative Diseases: Basic and Clinical Pharmacology.” Cerebellum 6, no. 4: 308–314. 17853088 10.1080/14734220601142878 PMC7102236

[brb370705-bib-0008] Avula, A. , Q. Bui , A. Kumar , et al. 2024. “Evaluating the Interaction Between Hemorrhagic Transformation and Cerebral Edema on Functional Outcome After Ischemic Stroke.” Journal of Stroke and Cerebrovascular Diseases 33, no. 10:107913. 39098362 10.1016/j.jstrokecerebrovasdis.2024.107913 PMC12045300

[brb370705-bib-0009] Baird, A. E. , G. A. Donnan , M. C. Austin , G. J. Fitt , S. M. Davis , and W. J McKay . 1994. “Reperfusion After Thrombolytic Therapy in Ischemic Stroke Measured by Single‐Photon Emission Computed Tomography.” Stroke 25, no. 1: 79–85. 8266387 10.1161/01.str.25.1.79

[brb370705-bib-0010] Berkhemer, O. A. , P. S. S. Fransen , D. Beumer , et al. 2015. “A Randomized Trial of Intraarterial Treatment for Acute Ischemic Stroke.” New England Journal of Medicine 372, no. 1: 11–20. 25517348 10.1056/NEJMoa1411587

[brb370705-bib-0011] Boulanger, J. M. , M. P. Lindsay , G. Gubitz , et al. 2018. “Canadian Stroke Best Practice Recommendations for Acute Stroke Management: Prehospital, Emergency Department, and Acute Inpatient Stroke Care, 6th Edition, Update 2018.” International Journal of Stroke 13, no. 9: 949–984. 30021503 10.1177/1747493018786616

[brb370705-bib-0012] Brekenfeld, C. , G. Schroth , P. Mordasini , et al. 2011. “Impact of Retrievable Stents on Acute Ischemic Stroke Treatment.” American Journal of Neuroradiology 32, no. 7: 1269–1273. 21566010 10.3174/ajnr.A2494 PMC7966059

[brb370705-bib-0013] Broocks, G. , and L Meyer . 2023. “New Advances in Diagnostic Radiology for Ischemic Stroke.” Journal of Clinical Medicine 12, no. 19: 6375. 37835019 10.3390/jcm12196375 PMC10573123

[brb370705-bib-0014] Chavda, V. , B. Chaurasia , A. Fiorindi , G. E. Umana , B. Lu , and N Montemurro . 2022. “Ischemic Stroke and SARS‐CoV‐2 Infection: The Bidirectional Pathology and Risk Morbidities.” Neurology International 14, no. 2: 391–405. 35645351 10.3390/neurolint14020032 PMC9149929

[brb370705-bib-0015] Chavda, V. , B. Chaurasia , K. Garg , et al. 2022. “Molecular Mechanisms of Oxidative Stress in Stroke and Cancer.” Brain Disorders 5: 100029.

[brb370705-bib-0016] Chen, J. , K. Duris , and X Yang . 2022. “Effect of Cerebral Microbleeds on Hemorrhagic Transformation and Functional Prognosis After Intravenous Thrombolysis of Cerebral Infarction.” Brain Hemorrhages 3, no. 3: 117–119.

[brb370705-bib-0017] Collins, T. J. , and P. J Samworth . 2008. “Therapeutic Hypothermia Following Cardiac Arrest: A Review of the Evidence.” Nursing in Critical Care 13, no. 3: 144–151. 18426470 10.1111/j.1478-5153.2008.00267.x

[brb370705-bib-0018] Contrada, M. , L. Pignolo, M. Vatrano, et al. 2024. “Application of Multidomain Cognitive Training in a Tele‐Neurorehabilitation Setting for Treatment of Post‐Stroke Cognitive Disorders.” Brain Sciences 15, no. 1: 11. 39851379 10.3390/brainsci15010011 PMC11764214

[brb370705-bib-0019] Copen, W. A. , P. W. Schaefer , and O Wu . 2011. “MR Perfusion Imaging in Acute Ischemic Stroke.” Neuroimaging Clinics of North America 21, no. 2: 259–283. 21640299 10.1016/j.nic.2011.02.007 PMC3135980

[brb370705-bib-0020] Costa Novo, J. , E. Rieffel , G. C. Velarde , et al. 2024. “Shorter Reperfusion Time in Stroke Is Associated With Better Cognition.” Canadian Journal of Neurological Sciences 51, no. 5: 644–649. 10.1017/cjn.2023.321 38052728

[brb370705-bib-0021] Diener, H. C. , K. R. Lees , P. Lyden , et al. 2008. “NXY‐059 for the Treatment of Acute Stroke: Pooled Analysis of the SAINT I and II Trials.” Stroke 39, no. 6: 1751–1758. 18369171 10.1161/STROKEAHA.107.503334

[brb370705-bib-0022] Dong, L. , E. Briceno , L. B. Morgenstern , and L. D Lisabeth . 2020. “Poststroke Cognitive Outcomes: Sex Differences and Contributing Factors.” Journal of the American Heart Association 9, no. 14:e016683. 32633589 10.1161/JAHA.120.016683 PMC7660722

[brb370705-bib-0023] Du, W. , J. Shen , and T. Su . 2023. “Neuroplasticity in Stroke Rehabilitation: Harnessing Brain's Adaptive Capacities for Enhanced Recovery.” Journal of Medical Research and Innovation 2, no. 11: 50–58.

[brb370705-bib-0024] Elkins, J. , R. Veltkamp , J. Montaner , et al. 2017. “Safety and Efficacy of Natalizumab in Patients With Acute Ischaemic Stroke (ACTION): A Randomised, Placebo‐Controlled, Double‐Blind Phase 2 Trial.” Lancet Neurology 16, no. 3: 217–226. 28229893 10.1016/S1474-4422(16)30357-X

[brb370705-bib-0025] Emberson, J. , K. R. Lees , P. Lyden , et al. 2014. “Effect of Treatment Delay, Age, and Stroke Severity on the Effects of Intravenous Thrombolysis With Alteplase for Acute Ischaemic Stroke: A Meta‐Analysis of Individual Patient Data From Randomised Trials.” Lancet 384, no. 9958: 1929–1935. 25106063 10.1016/S0140-6736(14)60584-5 PMC4441266

[brb370705-bib-0026] Enomoto, M. , A. Endo , H. Yatsushige , K. Fushimi , and Y Otomo . 2019. “Clinical Effects of Early Edaravone Use in Acute Ischemic Stroke Patients Treated by Endovascular Reperfusion Therapy.” Stroke. 50, no. 3: 652–658. 30741623 10.1161/STROKEAHA.118.023815

[brb370705-bib-0027] GBD 2019 Risk Factors Collaborators . 2020. “Global Burden of 87 Risk Factors in 204 Countries and territories, 1990–2019: A Systematic Analysis for the Global Burden of Disease Study 2019.” Lancet 396, no. 10258: 1223–1249. 33069327 10.1016/S0140-6736(20)30752-2 PMC7566194

[brb370705-bib-0028] GBD 2019 Stroke Collaborators . 2021. “Global, Regional, and National Burden of Stroke and Its Risk Factors, 1990–2019: A Systematic Analysis for the Global Burden of Disease Study 2019.” Lancet Neurology 20, no. 10: 795–820. 34487721 10.1016/S1474-4422(21)00252-0 PMC8443449

[brb370705-bib-0029] GBD 2019 Viewpoint Collaborators . 2020. “Five Insights From the Global Burden of Disease Study 2019.” Lancet 396, no. 10258: 1135–1159. 33069324 10.1016/S0140-6736(20)31404-5 PMC7116361

[brb370705-bib-0030] Goyal, M. , B. K. Menon , W. H. van Zwam , et al. 2016. “Endovascular Thrombectomy After Large‐Vessel Ischaemic Stroke: A Meta‐Analysis of Individual Patient Data From Five Randomised Trials.” Lancet 387, no. 10029: 1723–1731. 26898852 10.1016/S0140-6736(16)00163-X

[brb370705-bib-0031] Gunduz, M. E. , B. Bucak , and Z. Keser . 2023. “Advances in Stroke Neurorehabilitation.” Journal of Clinical Medicine 12, no. 21: 6734. 10.3390/jcm12216734. 37959200 PMC10650295

[brb370705-bib-0032] Hacke, W. , T. Brott , L. Caplan , et al. 1999. “Thrombolysis in Acute Ischemic Stroke: Controlled Trials and Clinical Experience.” Neurology 53: S3–S14. 10532643

[brb370705-bib-0033] Hou, Z. , and J. S. Brenner . 2024. “Developing Targeted Antioxidant Nanomedicines for Ischemic Penumbra: Novel Strategies in Treating Brain Ischemia‐Reperfusion Injury.” Redox Biology 73: 13. 10.1016/j.redox.2024.103185. PMC11127604 38759419

[brb370705-bib-0034] Humphrey, S. , K. E. Pike , B. Long , et al. 2024. “Neuropsychological Outcomes Following Endovascular Clot Retrieval and Intravenous Thrombolysis in Ischemic Stroke.” Journal of the International Neuropsychological Society 8, no. 30: 764–776. 10.1017/S1355617724000535 39410801

[brb370705-bib-0035] Jauch, E. C. , J. L. Saver , H. P. Adams , et al. 2013. “Guidelines for the Early Management of Patients With Acute Ischemic Stroke: A Guideline for Healthcare Professionals From the American Heart Association/American Stroke Association.” Stroke 44, no. 3: 870–947. 23370205 10.1161/STR.0b013e318284056a

[brb370705-bib-0036] Joundi, R. A. , E. E. Smith, J. Mandzia, et al. 2024. “Effect of Endovascular Thrombectomy for Acute Ischemic Stroke on Cognitive Outcomes: A Secondary Analysis of the ESCAPE Trial.” Neurology 102, no. 10:e209270. 38739880 10.1212/WNL.0000000000209270 PMC11177593

[brb370705-bib-0037] Kasemsap, N. , N. Vorasoot, K. Kongbunkiat, S. Tiamkao, W. Boonsawat , and K. Sawanyawisuth . 2022. “Factors Associated With Favorable Outcomes in Acute Severe Stroke Patients: A Real‐World, National Database Study.” Biomedical Reports 17, no. 3: 74. 10.3892/br.2022.1557. 35950096 PMC9353649

[brb370705-bib-0038] Khoy, K. , D. Mariotte , G. Defer , G. Petit , O. Toutirais , and B Le Mauff . 2020. “Natalizumab in Multiple Sclerosis Treatment: From Biological Effects to Immune Monitoring.” Frontiers in Immunology 11: 549842. 33072089 10.3389/fimmu.2020.549842 PMC7541830

[brb370705-bib-0039] Kleinbongard, P. , S. Gent , A. Skyschally , and G. Heusch . 2016. “Infarct Size Reduction by Remote Ischemic Perconditioning in Pigs Requires Activation of the SAFE but Not the RISK Pathway.” Supplement, Faseb Journal 30, no. S1: 1207.1. 10.1096/fasebj.30.1_supplement.1207.1.

[brb370705-bib-0040] Kovacs, A. , A. Mathe , and E. Frecska . 2024. “The Potential Use of Dimethyltryptamine Against Ischemia‐Reperfusion Injury of the Brain.” Journal of Neuroscience and Neurological Disorders 8: 050–056. 10.29328/journal.jnnd.1001097.

[brb370705-bib-0041] Kwon, H. S. , and S. H Koh . 2020. “Neuroinflammation in Neurodegenerative Disorders: The Roles of Microglia and Astrocytes.” Translational Neurodegeneration 9, no. 1: 42. 33239064 10.1186/s40035-020-00221-2 PMC7689983

[brb370705-bib-0042] Lampl, Y. , M. Boaz , R. Gilad , et al. 2007. “Minocycline Treatment in Acute Stroke: An Open‐Label, Evaluator‐Blinded Study.” Neurology 69, no. 14: 1404–1410. 17909152 10.1212/01.wnl.0000277487.04281.db

[brb370705-bib-0043] Lees, K. R. , J. A. Zivin , T. Ashwood , et al. 2006. “NXY‐059 for Acute Ischemic Stroke.” New England Journal of Medicine 354, no. 6: 588–600. 16467546 10.1056/NEJMoa052980

[brb370705-bib-0044] Li, J. , W. Cao , F. Zhao , and P Jin . 2024. “Cost‐Effectiveness of Edaravone Dexborneol Versus dl‐3‐*n*‐Butylphthalide for the Treatment of Acute Ischemic Stroke: A Chinese Health Care Perspective.” BMC Public Health [Electronic Resource] 24, no. 1: 436. 38347500 10.1186/s12889-024-17959-3 PMC10860239

[brb370705-bib-0045] Liang, H. , X. Wang , X. Quan , et al. 2023. “Different Doses of Tenecteplase vs. Alteplase for Acute Ischemic Stroke Within 4.5 Hours of Symptom Onset: A Network Meta‐Analysis of Randomized Controlled Trials.” Frontiers in Neurology 14: 1176540. 10.3389/fneur.2023.1176540. 37333014 PMC10274135

[brb370705-bib-0046] Lin, B. , M. Wang , X. Chen , L. Chai , J. Ni , and J. Huang . 2024. “Involvement of P2×7R‐Mediated Microglia Polarization and Neuroinflammation in the Response to Electroacupuncture on Post‐Stroke Memory Impairment.” Brain Research Bulletin 212: 110967. 38670470 10.1016/j.brainresbull.2024.110967

[brb370705-bib-0047] Lin, L. , X. Wang , and Z. Yu . 2016. “Ischemia‐Reperfusion Injury in the Brain: Mechanisms and Potential Therapeutic Strategies.” Biochemistry & Pharmacology: Open Access 5, no. 4: 213. 10.4172/2167-0501.1000213. 29888120 PMC5991620

[brb370705-bib-0048] Liu, X. , C. Xie , Y. Wang , et al. 2024. “Ferritinophagy and Ferroptosis in Cerebral Ischemia Reperfusion Injury.” Neurochemical Research 49, no. 8: 1965–1979. 38834843 10.1007/s11064-024-04161-5 PMC11233298

[brb370705-bib-0049] Luqman, S. , A. Mumtaz, S. Kulsoom, S. Ahmed , A. Malik , and S. A. Haider . 2023. “Predictors of Mortality and Poor Functional Outcome in Patients With Acute Stroke Admitted to a Tertiary Care Hospital in Pakistan.” Biological and Clinical Sciences Research Journal 2023, no. 1: 281.

[brb370705-bib-0050] Marasini, S. , and X. Jia . 2024. “Neuroprotective Approaches for Brain Injury after Cardiac Arrest: Current Trends and Prospective Avenues.” Journal of Stroke 26, no. 2: 203–230. 38836269 10.5853/jos.2023.04329 PMC11164592

[brb370705-bib-0051] Mehla, J. , S. H. Deibel , H. Karem , et al. 2023. “Repeated Multi‐Domain Cognitive Training Prevents Cognitive Decline, Anxiety and Amyloid Pathology Found in a Mouse Model of Alzheimer Disease.” Communications Biology 6, no. 1: 1145. 37950055 10.1038/s42003-023-05506-6 PMC10638434

[brb370705-bib-0052] Miotto, G. , M. Rossetto , M. L. Di Paolo , et al. 2020. “Insight Into the Mechanism of Ferroptosis Inhibition by Ferrostatin‐1.” Redox Biology 28: 101328. 31574461 10.1016/j.redox.2019.101328 PMC6812032

[brb370705-bib-0053] Mokin, M. , C. T. Primiani , A. H. Siddiqui , and A. S Turk . 2017. “ASPECTS (Alberta Stroke Program Early CT Score) Measurement Using Hounsfield Unit Values When Selecting Patients for Stroke Thrombectomy.” Stroke. 48, no. 6: 1574–1579. 28487329 10.1161/STROKEAHA.117.016745

[brb370705-bib-0054] Myers, S. J. , V. Agapova , S. V. Patel , et al. 2023. “Acute Minocycline Treatment Inhibits Microglia Activation, Reduces Infarct Volume, and Has Domain‐Specific Effects on Post‐Ischemic Stroke Cognition in Rats.” Behavioural Brain Research 455: 114680. 37742808 10.1016/j.bbr.2023.114680

[brb370705-bib-0055] Nagaraja, N. , A. Farooqui , A. B. Zahid , and S Kaur . 2021. “Factors Associated With the Presence of Cerebral Microbleeds and Its Influence on Outcomes of Stroke Not Treated With Alteplase.” Clinical Neurology and Neurosurgery 207: 106798. 34252690 10.1016/j.clineuro.2021.106798 PMC9020607

[brb370705-bib-0056] Nogueira, R. G. , A. P. Jadhav , D. C. Haussen , et al. 2018. “Thrombectomy 6 to 24 H After Stroke With a Mismatch Between Deficit and Infarct.” New England Journal of Medicine 378, no. 1: 11–21. 29129157 10.1056/NEJMoa1706442

[brb370705-bib-0057] Ospel, J. M. , L. Rinkel, A. Ganesh, et al. 2024. “Influence of Infarct Morphology and Patterns on Cognitive Outcomes after Endovascular Thrombectomy.” Stroke 55: 1349–1358. 10.1161/STROKEAHA.123.045825. 38511330

[brb370705-bib-0058] Palmer, S. J 2023. “Identification, Care and Prevention of Stroke Is Possible.” British Journal of Healthcare Assistants 17, no. 6: 236–239.

[brb370705-bib-0059] Patel, R. , S. Ispoglou , and S Apostolakis . 2014. “Desmoteplase as a Potential Treatment for Cerebral Ischaemia.” Expert Opinion on Investigational Drugs 23, no. 6: 865–873. 24766516 10.1517/13543784.2014.911285

[brb370705-bib-0060] Pedre, B. , U. Barayeu , D. Ezeriņa , and T. P Dick . 2021. “The Mechanism of Action of *N*‐Acetylcysteine (NAC): The Emerging Role of H2S and Sulfane Sulfur Species.” Pharmacology & Therapeutics 228: 107916. 34171332 10.1016/j.pharmthera.2021.107916

[brb370705-bib-0061] Pexman, J. H. W. , P. A. Barber , M. D. Hill , et al. 2001. “Use of the Alberta Stroke Program Early CT Score (ASPECTS) for Assessing CT Scans in Patients With Acute Stroke.” American Journal of Neuroradiology 22, no. 8: 1534–1542. 11559501 PMC7974585

[brb370705-bib-0062] Powers, W. J. , A. A. Rabinstein , T. Ackerson , et al. 2019. “Guidelines for the Early Management of Patients with Acute Ischemic Stroke: 2019 Update to the 2018 Guidelines for the Early Management of Acute Ischemic Stroke: A Guideline for Healthcare Professionals from the American Heart Association/American Stroke Association.” Stroke 50, no. 12: e344–e418. 31662037 10.1161/STR.0000000000000211

[brb370705-bib-0063] Ramnarine, I. V. P. , O. W. Rasheed, P. J. Laud, A. Majid, K. A. Harkness , and S. M. Bell . 2023. “Thrombolysis Outcomes in Acute Ischaemic Stroke Patients With Pre‐Existing Cognitive Impairment.” Life 13, no. 4: 1055–1055. 37109584 10.3390/life13041055 PMC10141004

[brb370705-bib-0064] Roaldsen, M. B. , H. Lindekleiv , and E. B Mathiesen . 2021. “Intravenous Thrombolytic Treatment and Endovascular Thrombectomy for Ischaemic Wake‐Up Stroke.” Cochrane Database of Systematic Reviews 12, no. 12:CD010995. 34850380 10.1002/14651858.CD010995.pub3 PMC8632645

[brb370705-bib-0065] Salim, H. , B. Musmar , N. Adeeb , et al. 2024. “Outcomes of Mechanical Thrombectomy in Anticoagulated Patients With Acute Distal and Medium Vessel Stroke.” European Stroke Journal 9, no. 4: 896–906. 38726983 10.1177/23969873241249295 PMC11569456

[brb370705-bib-0066] Savaliya, R. , V. Chavda , B. Patel , R. Brahmbhatt , E. G. Figueiredo , and B Chaurasia . 2024a. “Post‐Ischemic Scars and “the Micro‐Metabolic‐Glia‐Cerebral Changes”: Do We Know Everything?” Neurosurgical Review 47, no. 1: 455. 39168927 10.1007/s10143-024-02721-5

[brb370705-bib-0067] Savaliya, R. , V. K. Chavda , B. Patel , R. Brahmbhatt , and B Chaurasia . 2024b. “Acute Ischemic Stroke: Research Perspective vs. Clinical Practice.” Neurosurgical Review 47, no. 1: 612. 39271530 10.1007/s10143-024-02853-8

[brb370705-bib-0068] Savitz, S. I. , and W. R Schäbitz . 2008. “A Critique of SAINT II.” Stroke 39, no. 4: 1389–1391. 18309145 10.1161/STROKEAHA.107.504415

[brb370705-bib-0069] Shinohara, Y. , I. Saito , S. Kobayashi , and S Uchiyama . 2009. “Edaravone (Radical Scavenger) Versus Sodium Ozagrel (Antiplatelet Agent) in Acute Noncardioembolic Ischemic Stroke (EDO Trial).” Cerebrovascular Diseases 27, no. 5: 485–492. 19321945 10.1159/000210190

[brb370705-bib-0070] Shirley, R. , E. N. J. Ord , and L. M Work . 2014. “Oxidative Stress and the Use of Antioxidants in Stroke.” Antioxidants. 3, no. 3: 472–501. 26785066 10.3390/antiox3030472 PMC4665418

[brb370705-bib-0071] Shuaib, A. , K. R. Lees , P. Lyden , et al. 2007. “NXY‐059 for the Treatment of Acute Ischemic Stroke.” New England Journal of Medicine 357, no. 6: 562–571. 17687131 10.1056/NEJMoa070240

[brb370705-bib-0072] Sloane, K. L. , and R. H. Hamilton . 2024. “Transcranial Direct Current Stimulation to Ameliorate Post‐Stroke Cognitive Impairment.” Brain Sciences 14, no. 6: 614–614. 38928614 10.3390/brainsci14060614 PMC11202055

[brb370705-bib-0073] Sumii, T. , and E. H Lo . 2002. “Involvement of Matrix Metalloproteinase in Thrombolysis‐Associated Hemorrhagic Transformation After Embolic Focal Ischemia in Rats.” Stroke 33, no. 3: 831–836. 11872911 10.1161/hs0302.104542

[brb370705-bib-0074] Sun, M. S. , H. Jin , and X. Sun , et al. 2018. “Free Radical Damage in Ischemia‐Reperfusion Injury: An Obstacle in Acute Ischemic Stroke After Revascularization Therapy.” Oxidative Medicine and Cellular Longevity 2018: 3804979. 29770166 10.1155/2018/3804979 PMC5892600

[brb370705-bib-0075] Tawil, S. E. , and K. W Muir . 2017. “Thrombolysis and Thrombectomy for Acute Ischaemic Stroke.” Clinical Medicine 17, no. 2: 161–165. 28365630 10.7861/clinmedicine.17-2-161 PMC6297620

[brb370705-bib-0076] Tian, X. , X. Li , M. Pan , L. Z. Yang , Y. Li , and W Fang . 2024. “Progress of Ferroptosis in Ischemic Stroke and Therapeutic Targets.” Cellular and Molecular Neurobiology 44, no. 1: 25. 38393376 10.1007/s10571-024-01457-6 PMC10891262

[brb370705-bib-0077] Turc, G. , P. Bhogal , U. Fischer , et al. 2019. “European Stroke Organisation (ESO)—European Society for Minimally Invasive Neurological Therapy (ESMINT) Guidelines on Mechanical Thrombectomy in Acute Ischaemic Stroke Endorsed by Stroke Alliance for Europe (SAFE).” European Stroke Journal 4, no. 1: 6–12. 31165090 10.1177/2396987319832140 PMC6533858

[brb370705-bib-0078] Wang, L. , Z. Zhang , and H Wang . 2021. “Naringin Attenuates Cerebral Ischemia‐Reperfusion Injury in Rats by Inhibiting Endoplasmic Reticulum Stress.” Translational Neuroscience 12, no. 1: 190–197. 34046215 10.1515/tnsci-2020-0170 PMC8134799

[brb370705-bib-0079] Xia, J. , T. Zhang, Y. Sun, et al. 2024. “Suppression of Neuronal CDK9/p53/VDAC Signaling Provides Bioenergetic Support and Improves Post‐Stroke Neuropsychiatric Outcomes.” Cellular and Molecular Life Sciences 81, no. 1: 384. 10.1007/s00018-024-05428-4. 39235466 PMC11377386

[brb370705-bib-0080] Yang, C. , J. Mo , and Q. Liu . 2024. “TXNIP/NLRP3 Aggravates Global Cerebral Ischemia‐Reperfusion Injury‐Induced Cognitive Decline in Mice.” Heliyon 10: e27423. 10.1016/j.heliyon.2024.e27423. 38496898 PMC10944238

[brb370705-bib-0081] Ye, Z. , R. Liu, and H. Wang, et al. 2024. “Neuroprotective Potential for Mitigating Ischemia Reperfusion‐Induced Damage.” Neural Regeneration Research 20: 2199–2217. 10.4103/NRR.NRR-D-23-01985. 39104164 PMC11759025

[brb370705-bib-0082] Yu, Z. , L. Lin , and X. Wang . 2017 “Pathophysiology of Ischemia‐Reperfusion Injury and Hemorrhagic Transformation in the Brain.” In Primer on Cerebrovascular Diseases. 2nd ed. Edited by L. R. Caplan , M. C. Leary , A. J. Thomas , et al., 121–124. Academic Press.

[brb370705-bib-0083] Zhang, L. , X. Y. Bai , K. Y. Sun , et al. 2024. “A New Perspective in the Treatment of Ischemic Stroke: Ferroptosis.” Neurochemical Research 49, no. 4: 815–833. 38170383 10.1007/s11064-023-04096-3

[brb370705-bib-0084] Zhang, L. , Y. L. Luo , Y. Xiang , et al. 2024. “Ferroptosis Inhibitors: Past, Present and Future.” Frontiers in Pharmacology 15: 1407335. 10.3389/fphar.2024.1407335. 38846099 PMC11153831

[brb370705-bib-0085] Zhang, T. L. , Z. W. Zhang , and W. Lin , et al. 2023. “Reperfusion After Hypoxia‐Ischemia Exacerbates Brain Injury With Compensatory Activation of the Anti‐Ferroptosis System: Based on a Novel Rat Model.” Neural Regeneration Research 18, no. 10: 2229–2236. 37056142 10.4103/1673-5374.369117 PMC10328270

[brb370705-bib-0086] Zhou, Y. , Y. He , S. Yan , et al. 2023. “Reperfusion Injury Is Associated with Poor Outcome in Patients With Recanalization after Thrombectomy.” Stroke 54, no. 1: 96–104. 36367100 10.1161/STROKEAHA.122.039337

